# The association between workplace violence and physicians’ and nurses’ job satisfaction in Macau

**DOI:** 10.1371/journal.pone.0207577

**Published:** 2018-12-05

**Authors:** Teris Cheung, Paul H. Lee, Paul S. F. Yip

**Affiliations:** 1 School of Nursing, Hong Kong Polytechnic University, Hong Kong SAR; 2 Centre for Suicide Research and Prevention, University of Hong Kong, Hong Kong SAR; University of Toronto, CANADA

## Abstract

**Background:**

This paper describes the association between workplace violence and job satisfaction among physicians and nurses in Macau. Convenience sampling was sourced from six health centers under the Macau Health Bureau.

**Methods:**

This study uses a cross-sectional self-administrative survey. The study used case studies research instruments for workplace violence in the health sector by country (from the ILO, ICN, WHO, PSI), the Minnesota Satisfaction Questionnaire and Perceived Stress Scale. The data collection period spanned from August to December, 2014.

**Data analysis:**

Multiple logistic regression examines levels of intrinsic and extrinsic satisfaction in physicians and nurses and significant correlates affecting their job satisfaction.

**Results:**

A total of 720 (14.9% physicians) participants were recruited. 57.2% of participants reported physical and psychological workplace violence in the preceding year. The most common forms of workplace violence were verbal abuse (53.4%), physical assault (16.1%), bullying / harassment (14.2%), sexual harassment (4.6%) and racial harassment (2.6%). Nurses were at a significantly higher risk of physical assault and verbal abuse compared to physicians. Patients, patients’ relatives, and colleagues were the main perpetrators. Worry about WPV, on-call duty and shift work, experience of bullying and verbal abuse and employment sector emerged as significant correlates affecting the intrinsic and extrinsic job satisfaction of physicians and nurses. Frontline staff, aged 30 and 39, coming from an ethnic minority, and perceived stress were significant correlates affecting nurses’ job satisfaction.

**Conclusions:**

WPV remains a significant concern in healthcare settings in Macau. Stakeholders should legally enforce a zero-tolerance policy towards WPV within healthcare workplaces. WPV is detrimental to healthcare professionals’ mental wellbeing, risking irreversible physical and psychological harm for its victims.

## 1. Introduction

The National Institute for Occupational Safety and Health (NIOSH) defines WPV workplace violence as “violent acts (including physical assaults and threats of assaults) directed toward persons at work or on duty.” [1]. In 2014, the American Nurses Association further included lateral violence (acts between colleagues, bullying, hostility, abuse of authority, and sexual harassment) in the definition of WPV.[2] 2013 saw a reported 25,630 incidents of WPV in the US, of which 74% apparently occurred in healthcare settings.[3] Workplace violence committed against medical occupational groups represented 10.2% of all workplace violence incidents. Research has consistently reported that violence incidence rates in public healthcare settings are higher than in the private health sector. [3] Workplace violence remains a threat in healthcare setting across America [3–6], United Kingdom [7], Italy [8], Norway [9], Jordan [10, 11], Australia [12], South Africa [13], Ghana [14], Japan [15], Macau [16], and Hong Kong. [17, 18] Extensive studies have investigated the risk factors for WPV and national workplace violence prevention guidelines have been put in place. Despite concerted effort has been made in any national or local setting to examine the psychological impact of WPV on physicians and nurses, the association between the (presumably) negative psychological impact of WPV and physicians’ and nurses’ job satisfaction has yet to be measured in any satisfactorily comprehensive way. Job dissatisfaction stemming from WPV may lead to professionals’ burnout [4, 19, 20], poor morale [21], increased likelihood to quit the job [22–25], diminished quality of care and increased psychiatric morbidity. [4, 25–29] Job satisfaction has also been found to be significantly correlated with depression in nurses. [30] To the author’s knowledge, no study has yet measured the association between WPV and job satisfaction among physicians and nurses specifically in Macau—a gap the study fills.

## 2. Materials and methods

### 2.1. Aim

This paper describes the association between workplace violence and job satisfaction among physicians and nurses in Macau. It further examines the risk factors for intrinsic and extrinsic job satisfaction among these professionals.

### 2.2. Study design

This study used a cross-sectional survey design.

### 2.3. Participants

Convenience sampling was recruited from six health centers under the Macau Health Bureau, namely, Coloane Health Center, Coloane Shek Pai Wan Temporary Health Center, Taipa Elderly Macau University of Science and Technology Hospital, the Macau Sino-Portuguese Nurses Association, the Macau Association of Medical Volunteers, and the Macau Surgical Association. As at 30 June 2014, Macau’s population stood at 636,200 (51% of whom were female). In 2014, there were 3582 healthcare professionals (1592 physicians and 1990 nurses) [31] working in the public or private health sector. Physicians and nurses, both male and female, aged between 20 and 40 or above, who had worked for these organizations for at least a year were invited to participate in this study. Participants were required to sign a written informed consent form attached on the questionnaire. Collaborating associations helped advertising and recruitment of participants. Registered nurses working in these health centers were also recruited to distribute the questionnaire to their colleagues to participate in this study. Part-time master students were posted in the communal facilities (e.g., staff canteens, library, hospital compound) to distribute questionnaire to potential participants. A gentle reminder was written on the front page of the questionnaire to avoid double-entry of the questionnaire.

### 2.4. Instruments

All questions in the survey were derived from the “Workplace Violence in the Health Sector: Country Case Studies Research Instruments Survey Questionnaire”.[32] The instrument was translated into Chinese, and we invited 8 mental health experts in Hong Kong to evaluate its content validity, including the appropriateness of the translation and comprehensibility of the questions asked. Confirmation of test-retest reliability (0.85) and consistency was assessed for the survey with 20 nurses in four regional hospitals in Hong Kong. A retest was performed two weeks later. The questions were then back-translated to English to verify the accuracy of the Chinese version. [33] This questionnaire comprises five domains. In the first, it gathers socio-demographic information (e.g. the gender, age and marital status of respondents). Next, it notes incidences of physical assault, capturing the perpetrators of abuse, its apparent motivations and management. Third, it records other kinds of abuse, including verbal abuse, bullying or harassment. The fourth section asks what health policy is intended to deal with these outbreaks, and the last canvasses suggestions for the prevention of WPV.

Additionally, the Minnesota Satisfaction Questionnaire (MSQ) [34] and the Chinese version of the Perceived Stress Scale (PSS) [35] were used to measure the level of job satisfaction and self-perceived level of stress among physicians and nurses in Macau, respectively. The MSQ consists of 20 short descriptive statements. Participants rate each item relating to their job satisfaction according to a 5-point Likert scale (1 = Very dissatisfied; 2 = Dissatisfied; 3 = Neutral, 4 = Satisfied, and 5 = Very Satisfied). The MSQ consists of two subscales, one devoted to intrinsic (item 1,2,3,4,7–11, 15–16, and 20) and the other to extrinsic job satisfaction (item 5–6, 12–14, and 19). For this study, Cronbach’s alpha for the intrinsic and extrinsic job satisfaction of the MSQ was taken as 0.957 and 0.946 respectively. The median was used as a cut-off value to classify respondents’ job satisfaction levels into low or high. Participants with a cut off score of ≥ 35 in intrinsic job satisfaction and ≥ 15 in extrinsic job satisfaction were considered satisfied in their job. Intrinsic and extrinsic job satisfactions were separately analyzed for physicians and nurses.

PSS comprises 14 questions eliciting participants’ thoughts and feelings over the last month on a 5-point Likert Scale (0–4), with 0 indicating they had a certain thought ‘Never’ and 4 that they entertained it ‘Very often’. PSS scores were obtained by re-versing the scores on the seven positive items, e.g., 0 = 4, 1 = 3, 2 = 2, etc., then summing across all 14 items. Items 4, 5, 6, 7, 9, 10, and 13 state a positive affect. Participants with a cut off score of ≥ 26 on the Perceived Stress Scales (PSS) were considered having disorders as referenced. The Cronbach’s alpha for PSS in the study came in at 0.818.

In this paper, the definition of WPV incorporated the definition suggested by the World Health Organization (Box 1) as below:

**Box 1**

Verbal abuse—vulgarity, insults, sniggering

Bullying—unreasonable workloads or shifts

Physical abuse—physical assault, slapping, kicking, other forms of physical affront

Sexual harassment—verbal remarks of a sexual nature, lewd gestures or hints, any form of sexualized physical action

### 2.5. Statistical analysis

Statistical analyses were performed using the statistical software SPSS 24.0. Frequency distributions for categorical data were calculated for those with high and low job satisfaction among the study cohort of physicians and nurses. A chi-squared test was performed to examine their differences. Univariate analysis generated crude odds ratios (COR) for participants characterised within single parameters having good (or bad) job satisfaction and served as the basis for bivariate logistic regressions. All socio-demographic variables with a p value of < 0.25 in the bivariate analysis were selected for multivariate logistic regression analysis. This screening criterion of a level of 0.25 for variable selection was based on Hosmer and Lemeshow’s [36] recommendation not to leave out potentially important covariates that may have been missed by univariate analysis. The multiple logistic regressions employed a procedure of selecting by backwards elimination. A fit model was created using significant background variables as well as variables indicating worries about workplace violence (WPV), perceived stress and certain types of experience with WPV. The study then assessed the adjusted association between risk factors and job satisfaction, taking account of background variables that were found to be significant in the multivariate analysis. Adjusted odds ratios (AOR) were derived and 95% confidence intervals (CIs) for associated factors calculated. All tests were two-tailed and had a significance level of 0.05.

### 2.6 Ethics approval

The study was approved by the Human Subjects Ethics Committee, and the Institutional Review Board of the Hong Kong Polytechnic University. Reference No: HSEARS20140417004.

## 3. Results

### 3.1. Socio-demographic, clinical and other characteristics of the sample population

A total of 900 questionnaires were distributed and 720 physicians and nurses were recruited to the study (14.9% doctors & 85.1% nurses). A total of 706 participants (104 physicians, 602 nurses) were included in the statistical analysis as 14 participants did not give their responses on the MSQ, excluding themselves from statistical analysis. 56.3% were married and 40.7% single. The majority of respondents (78.8%) were female. Approximately every 1 in 7 physicians and 1 in 30 nurses in Macau participated in this study. The studyʼs response rate was 80%.

Table 1 reports the frequency distribution of the study’s physicians by their socio-demographic characteristics. 64.4% were aged between 20 and 39, with 42.3% in their 30s (between 30 and 39 years old). Two-thirds were male and 73.1% married. The majority of participants were frontline staff (97.1%) working on a full-time basis (95.2%). Over half the participants worked on shift (58.7%), being on call between 6 pm and 7 am (55.8%). Nearly 60% of participants had ≤ 10 years of clinical experience. 55.8% worked for public organizations, 32.7% private organizations and 11.5% some form of non-profit organization.

**Table 1:** Socio-demographic characteristics—Physicians.

	Total		Low		High			Low		High		
			Intrinsic-satisfiers		Intrinsic-satisfiers			Extrinsic-satisfiers		Extrinsic-satisfiers		
	N = 104		N = 51		N = 53		λ^2^^a^	N = 52		N = 52		λ^2^^a^
	N (%)		N (%)		N (%)			N (%)		N (%)		
Age Group							6.437*					5.116^+^
20–29	23	(22.1)	14	(27.5)	9	(17.0)		13	(25.0)	10	(19.2)	
30–39	44	(42.3)	25	(49.0)	19	(35.8)		26	(50.0)	18	(34.6)	
40 and over	37	(35.6)	12	(23.5)	25	(47.2)		13	(25.0)	24	(46.2)	
Gender							2.536					1.077
Female	35	(33.7)	21	(41.2)	14	(26.4)		20	(38.5)	15	(28.8)	
Male	69	(66.3)	30	(58.8)	39	(73.6)		32	(61.5)	37	(71.2)	
Marital status							0.104					0.782
Single/Separated/Divorced/Widow	28	(26.9)	14	(27.5)	14	(26.4)		12	(23.1)	16	(30.8)	
Married	76	(73.1)	37	(72.5)	39	(73.6)		40	(76.9)	36	(69.2)	
Identify themselves in workplace							3.776^+^					5.699*
Minority	87	(83.7)	39	(76.5)	48	(90.6)		39	(75.0)	48	(92.3)	
Majority	17	(16.3)	12	(23.5)	5	(9.4)		13	(25.0)	4	(7.7)	
Frontline staff							1.924					3.089^b^
Yes	101	(97.1)	50	(98.0)	51	(96.2)		49	(94.2)	52	(100.0)	
No	3	(2.9)	1	(2.0)	2	(3.8)		3	(5.8)	0	(.0)	
Working experience in healthcare sector							9.870*					2.651
5 years or less	40	(38.5)	23	(45.1)	17	(32.1)		22	(42.3)	18	(34.6)	
6–10 years	22	(21.2)	15	(29.4)	7	(13.2)		13	(25.0)	9	(17.3)	
More than 10 years	42	(40.3)	13	(25.5)	29	(54.7)		17	(32.7)	25	(48.1)	
Employment sector							14.726**					3.940
Private organization	34	(32.7)	25	(49.0)	9	(17.0)		21	(40.4)	13	(25.0)	
Non-profit organization	12	(11.5)	7	(13.7)	5	(9.4)		7	(13.5)	5	(9.6)	
Public organization	58	(55.8)	19	(37.3)	39	(73.6)		24	(46.2)	34	(65.4)	
Employment status							0.253					0.210^b^
Full-time	99	(95.2)	48	(94.1)	51	(96.2)		49	(94.2)	50	(96.2)	
Part-time/Temporary/Casual	5	(4.8)	3	(5.9)	2	(3.8)		3	(5.8)	2	(3.8)	
Shifts duty							0.001					0.991
No	43	(41.3)	21	(41.2)	22	(41.5)		19	(36.5)	24	(46.2)	
Yes	61	(58.7)	30	(58.8)	31	(58.5)		33	(63.5)	28	(53.8)	
Working on call between 6 pm and 7 am							1.020					2.495
No	46	(44.2)	20	(39.2)	26	(49.1)		19	(36.5)	27	(51.9)	
Yes	58	(55.8)	31	(60.8)	27	(50.9)		33	(63.5)	25	(48.1)	
Direct patient care							0.001					2.041^b^
Yes	98	(98.0)	48	(98.0)	50	(98.0)		48	(96.0)	50	(100.0)	
No	2	(2.0)	1	(2.0)	1	(2.0)		2	(4.0)	0	(.0)	
Number of staff present in the same work setting							0.808					0.253
5 or less	56	(54.4)	30	(58.8)	26	(50.0)		29	(56.9)	27	(51.9)	
More than 5	47	(45.6)	21	(41.2)	26	(50.0)		22	(43.1)	25	(48.1)	

^+^ p < 0.01;

* p < 0.05;

** p < 0.005

^a^ Chi-square test;

^b^ Fisher Exact Test

Table 2 comparably reports the frequency distribution of nursing staff by socio-demographic characteristics. 63.3% were aged between 20 and 39, 42.4% of whom were in their 20s (between 20 and 29 years old). The majority of participants (86.5%) were female; 54.5% were married. Most participants were frontline staff (96.5%) working full-time (97.8%). Over 70% of the participants did shift work (78.4%) and were on call between 6 pm. and 7 am. (73.4%). 63.4% had ≤ 10 years of clinical experience. 69.3% worked in public organizations, 18.8% in private organizations and 12% for non-profits.

**Table 2:** Socio-demographic characteristics—Nurses.

	Total		Low		High			Low		High		
			Intrinsic-satisfiers		Intrinsic-satisfiers			Extrinsic-satisfiers		Extrinsic-satisfiers		
	N = 602		N = 271		N = 331		λ^2^^a^	N = 288		N = 314		λ^2^^a^
	N (%)		N (%)		N (%)			N (%)		N (%)		
Age Group							5.249^+^					14.780**
20–29	255	(42.4)	117	(43.2)	138	(41.7)		128	(44.4)	127	(40.4)	
30–39	186	(30.9)	93	(34.3)	93	(28.1)		103	(35.8)	83	(26.4)	
40 and over	161	(26.7)	61	(22.5)	100	(30.2)		57	(19.8)	104	(33.1)	
Gender							1.767					1.032
Female	521	(86.5)	229	(84.5)	292	(88.2)		245	(85.1)	276	(87.9)	
Male	81	(13.5)	42	(15.5)	39	(11.8)		43	(14.9)	38	(12.1)	
Marital status							0.074					1.683
Single/Separated/Divorced/Widow	274	(45.5)	125	(46.1)	149	(45.0)		139	(48.3)	135	(43.0)	
Married	328	(54.5)	146	(53.9)	182	(55.0)		149	(51.7)	179	(57.0)	
Identify themselves in workplace							4.777*					1.278
Minority	94	(15.6)	52	(19.2)	42	(12.7)		50	(17.4)	44	(14.0)	
Majority	508	(84.4)	219	(80.8)	289	(87.3)		238	(82.6)	270	(86.0)	
Frontline Staff							11.074**					1.835
Yes	581	(96.5)	269	(99.3)	312	(94.3)		281	(97.6)	300	(95.5)	
No	21	(3.5)	2	(0.7)	19	(5.7)		7	(2.4)	14	(4.5)	
Working experience in healthcare sector							4.430					7.379*
5 years or less	262	(43.5)	124	(45.8)	138	(41.7)		132	(45.8)	130	(41.4)	
6–10 years	120	(19.9)	60	(22.1)	60	(18.1)		66	(22.9)	54	(17.2)	
More than 10 years	220	(36.5)	87	(32.1)	133	(40.2)		90	(31.3)	130	(41.4)	
Employment sector							9.923*					20.270**
Private organization	113	(18.8)	52	(19.2)	61	(18.4)		53	(18.4)	60	(19.1)	
Non-profit organization	72	(12.0)	20	(7.4)	52	(15.7)		17	(5.9)	55	(17.5)	
Public organization	417	(69.3)	199	(73.4)	218	(65.9)		218	(75.7)	199	(63.4)	
Employment status							0.419					1.552
Full-time	589	(97.8)	264	(97.4)	325	(98.2)		284	(98.6)	305	(97.1)	
Part-time/Temporary/Casual	13	(2.2)	7	(2.6)	6	(1.8)		4	(1.4)	9	(2.9)	
Shifts duty							10.820**					26.974**
No	130	(21.6)	42	(15.5)	88	(26.6)		36	(12.5)	94	(29.9)	
Yes	472	(78.4)	229	(84.5)	243	(73.4)		252	(87.5)	220	(70.1)	
Working on call between 6 pm and 7 am							16.686**					17.339**
No	160	(26.6)	50	(18.5)	110	(33.2)		54	(18.8)	106	(33.8)	
Yes	442	(73.4)	221	(81.5)	221	(66.8)		234	(81.3)	208	(66.2)	
Direct patient care							1.463					11.038**
Yes	581	(96.8)	265	(97.8)	316	(96.0)		286	(99.3)	295	(94.6)	
No	19	(3.2)	6	(2.2)	13	(4.0)		2	(.7)	17	(5.4)	
Number of staff present in the same work setting							0.022					0.195
5 or less	333	(55.3)	149	(55.0)	184	(55.6)		162	(56.3)	171	(54.5)	
More than 5	269	(44.7)	122	(45.0)	147	(44.4)		126	(43.8)	143	(45.5)	

^+^ p < 0.01;

* p < 0.05;

** p < 0.005

^a^ Chi-square Test

### 3.2. Prevalence of WPV

We have reported elsewhere about the prevalence of WPV towards physicians and nurses in Macau. [16] 57.2% of participants reported WPV in the preceding year (16.1% physical assault, 53.4% verbal abuse, 14.2% bullying/ harrassment, 4.6% sexual harrassment, and 2.6% racial harrassment). Patients, apart from family members and colleagues, were the main perpetrators for all kinds of WPV. Nurses reported a signficantly higher risk of physical assault and verbal abuse compared to physicians.

### 3.3. Intrinsic and extrinsic job satisfaction and perceived stress level among physicians and nurses

A total of 53 (51.0%) physicians can be accounted highly intrinsically satisfied and 52 (50.0%) highly extrinsically satisfied. Among those with low intrinsic satisfaction, 56.9% perceived themselves as highly stressed. For those with low extrinsic satisfaction, the corresponding rate for stress was 51.9% (Table 3).

**Table 3:** Worry about WPV, perceived stress and experience with WPV—Physicians.

	Total		Low		High			Low		High		
			Intrinsic-satisfiers		Intrinsic-satisfiers			Extrinsic-satisfiers		Extrinsic-satisfiers		
	N = 104		N = 51		N = 53		λ^2^^a^	N = 52		N = 52		λ^2^^a^
	N (%)		N (%)		N (%)			N (%)		N (%)		
Violence worried							6.510*					5.479^+^
Not worried	52	(50.0)	19	(37.3)	33	(62.3)		21	(40.4)	31	(59.6)	
A bit worried	29	(27.9)	18	(35.3)	11	(20.8)		15	(28.8)	14	(26.9)	
Worried	23	(22.1)	14	(27.5)	9	(17.0)		16	(30.8)	7	(13.5)	
Perceived stress level							5.503*					1.902
Low	57	(54.8)	22	(43.1)	35	(66.0)		25	(48.1)	32	(61.5)	
High	47	(45.2)	29	(56.9)	18	(34.0)		27	(51.9)	20	(38.5)	
Experience with physical attack in the last 12 months							0.002^b^					0.000^b^
No	98	(96.1)	49	(96.1)	49	(96.1)		49	(96.1)	49	(96.1)	
Yes	4	(3.9)	2	(3.9)	2	(3.9)		2	(3.9)	2	(3.9)	
Being physical attacked by^#^							NA					NA
Patients / Patients’ relatives	4	(100.0)	2	(100)	2	(100)		2	(100)	2	(100)	
Staff / Supervisor / Management	0	-	0	-	0	-		0	-	0	-	
General public / Others	0	-	0	-	0	-		0	-	0	-	
Experience with verbal abuse in the last 12 month							0.312					0.000^b^
No	64	(61.5)	30	(58.8)	34	(64.2)		32	(61.5)	32	(61.5)	
Yes	40	(38.5)	21	(41.2)	19	(35.8)		20	(38.5)	20	(38.5)	
Being verbal abuse by^#^							1.541^c^					1.830^c^
Patients / Patients’ relatives	35	(87.5)	18	(85.7)	17	(89.5)		17	(85.0)	18	(90.0)	
Staff / Supervisor / Management	5	(12.5)	3	(14.3)	2	(10.5)		3	(15.0)	2	(10.0)	
General public / Others	1	(2.5)	1	(4.8)	0	-		1	(5.0)	0	-	
Experience with being bullied/mobbed in the last 12 months							0.469^b^					3.391^+^
No	92	(88.5)	44	(86.3)	48	(90.6)		43	(82.7)	49	(94.2)	
Yes	12	(11.5)	7	(13.7)	5	(9.4)		9	(17.3)	3	(5.8)	
Being bullied/mobbed by^#^												
Patients / Patients’ relatives	8	(72.7)	4	(57.1)	4	(100.0)	4.352^c^	5	(62.5)	3	(100.0)	2.856^c^
Staff / Supervisor / Management	3	(27.3)	3	(42.9)	0	-		3	(37.5)	0	-	
General public / Others	0	-	0	-	0	-		0	-	0	-	
Experience with sexually harassment in the last 12 months							0.002^b^					0.000^b^
No	100	(96.2)	49	(96.1)	51	(96.2)		50	(96.2)	50	(96.2)	
Yes	4	(3.8)	2	(3.9)	2	(3.8)		2	(3.8)	2	(3.8)	
Being sexually harassment by^#^							NA					NA
Patients / Patients’ relatives	4	(100.0)	2	(100.0)	2	(100.0)		2	(100.0)	2	(100.0)	
Staff / Supervisor / Management	0	-	0	-	0	-		0	-	0	-	
General public / Others	0	-	0	-	0	-		0	-	0	-	
Experience with racially harassment in the last 12 months							0.962^a^					1.040^b^
No	100	(96.2)	50	(98.0)	50	(94.3)		51	(98.1)	49	(94.2)	
Yes	4	(3.8)	1	(2.0)	3	(5.7)		1	(1.9)	3	(5.8)	
Being racially harassment by^#^							NA					NA
Patients / Patients’ relatives	3	(75.0)	0	-	3	(100.0)		0	-	3	(100.0)	
Staff / Supervisor / Management	2	(25.0)	1	(100.0)	1	(33.3)		1	(100.0)	1	(33.3)	
General public / Others	0	-	0	-	0	-		0	-	0	-	

^+^ p < 0.01;

* p < 0.05;

** p < 0.005

^a^ Chi-square Test;

^b^ Fisher Exact Test;

^c^ First-order Rao-Scott Corrected Chi-square

^#^ Respondents can give more than one answer.

NA = Not available due to small sample

Among the nurses, 331 (55.0%) were highly intrinsically satisfied and 314 (52.2%) highly extrinsically satisfied. For those with low intrinsic satisfaction, 67.5% thought they were highly stressed. For those with low extrinsic satisfaction, 65.3% reported themselves as under this level of stress (Table 4).

**Table 4:** Worry about WPV, perceived stress and experience with WPV—Nurses.

	Total		Low		High			Low		High		
			Intrinsic-satisfiers		Intrinsic-satisfiers			Extrinsic-satisfiers		Extrinsic-satisfiers		
	N = 602		N = 271		N = 331		λ^2^^a^	N = 288		N = 314		λ^2^^a^
	N (%)		N (%)		N (%)			N (%)		N (%)		
Violence worried							36.018**					41.803**
Not worried	180	(29.9)	55	(20.3)	125	(37.8)		62	(21.5)	118	(37.6)	
A bit worried	186	(30.9)	76	(28.0)	110	(33.2)		75	(26.0)	111	(35.4)	
Worried	236	(39.2)	140	(51.7)	96	(29.0)		151	(52.4)	85	(27.1)	
Perceived stress level							28.956**					21.452**
Low	268	(44.5)	88	(32.5)	180	(54.4)		100	(34.7)	168	(53.5)	
High	334	(55.5)	183	(67.5)	151	(45.6)		188	(65.3)	146	(46.5)	
Experience with physical attack in the last 12 months							5.924*					15.242**
No	489	(81.6)	208	(77.3)	281	(85.2)		215	(75.2)	274	(87.5)	
Yes	110	(18.4)	61	(22.7)	49	(14.8)		71	(24.8)	39	(12.5)	
Being physical attacked by^#^							1.078^b^					2.867^b^
Patients / Patients’ relatives	106	(96.4)	60	(98.4)	46	(93.9)		70	(98.6)	36	(92.3)	
Staff / Supervisor / Management	5	(4.5)	2	(3.3)	3	(6.1)		2	(2.8)	3	(7.7)	
General public / Others	0	-	0	-	0	-		0	-	0	-	
Experience with verbal abuse in the last 12 months							14.802**					36.437**
No	261	(43.4)	94	(34.8)	167	(50.5)		88	(30.7)	173	(55.1)	
Yes	340	(56.6)	176	(65.2)	164	(49.5)		199	(69.3)	141	(44.9)	
Being verbal abuse by^#^							4.738^b^					3.474^b^
Patients / Patients’ relatives	295	(86.8)	152	(86.4)	143	(87.2)		171	(85.9)	124	(87.9)	
Staff / Supervisor / Management	63	(18.5)	35	(19.9)	28	(17.1)		38	(19.1)	25	(17.7)	
General public / Others	3	(0.9)	3	(1.7)	0	-		3	(1.5)	0	-	
Experience with being bullied/mobbed in the last 12 months							21.170**					14.250**
No	513	(85.2)	211	(77.9)	302	(91.2)		229	(79.5)	284	(90.4)	
Yes	89	(14.8)	60	(22.1)	29	(8.8)		59	(20.5)	30	(9.6)	
Being bullied/mobbed by^#^							5.310^b^					0.3489^b^
Patients / Patients’ relatives	62	(69.7)	46	(76.7)	16	(55.2)		41	(69.5)	21	(70.0)	
Staff / Supervisor / Management	32	(36.0)	18	(30.0)	14	(48.3)		21	(35.6)	11	(36.7)	
General public / Others	2	(2.2)	1	(1.7)	1	(3.4)		1	(1.7)	1	(3.3)	
Experience with sexually harassment in the last 12 months							0.000					5.449*
No	573	(95.2)	258	(95.2)	315	(95.2)		268	(93.1)	305	(97.1)	
Yes	29	(4.8)	13	(4.8)	16	(4.8)		20	(6.9)	9	(2.9)	
Being sexually harassment by^#^							1.534^b^					0.127^b^
Patients / Patients’ relatives	19	(65.5)	10	(76.9)	9	(56.3)		13	(65.0)	6	(66.7)	
Staff / Supervisor / Management	11	(37.9)	4	(30.8)	7	(43.8)		8	(40.0)	3	(33.3)	
General public / Others	0	-	0	-	0	-		0	-	0	-	
Experience with racially harassment in the last 12 months							0.430					0.186
No	587	(97.5)	263	(97.0)	324	(97.9)		280	(97.2)	307	(97.8)	
Yes	15	(2.5)	8	(3.0)	7	(2.1)		8	(2.8)	7	(2.2)	
Being racially harassment by^#^							1.407^b^					2.196^b^
Patients / Patients’ relatives	8	(53.3)	4	(50.0)	4	(57.1)		5	(62.5)	3	(42.9)	
Staff / Supervisor / Management	7	(46.7)	4	(50.0)	3	(42.9)		4	(50.0)	3	(42.9)	
General public / Others	1	(6.7)	1	(12.5)	0	-		0	-	1	(14.3)	

^+^ p < 0.01;

* p < 0.05;

** p < 0.005

^a^ Chi-square Test;

^b^ First-order Rao-Scott Corrected Chi-square.

^#^ Respondents can give more than one answer

### 3.4. Workplace violence and job satisfaction among physicians and nurses

#### 3.4.1. Physicians

Of physicians with low intrinsic satisfaction, 62.8% reported that they were “worried” or “a bit worried” about WPV. By contrast, only 37.8% of physicians with high intrinsic satisfaction reported the same levels of concern about WPV. Among those less extrinsically satisfied, 59.6% of physicians reported they were “a bit worried” or “worried” about WPV. Of the highly extrinsically satisfied physicians, 40.4% were “a bit worried” or “very worried” on the same subject. More physicians in the less extrinsically satisfied subgroup had been through the experience of being bullied than among the more satisfied (Table 3).

#### 3.4.2. Nurses

A total of 79.7% of the less extrinsically satisfied nurses reported “worried” or “a bit worried” about WPV. This is more than the 62.2% of highly intrinsically satisfied nurses reporting the same degree of worry. Among the less extrinsically satisfied subgroup, 78.4% were worried or a bit worried, whereas the corresponding rate among the highly extrinsically satisfied was 62.5%. The less extrinsically satisfied had been more frequently the object of physical attack, verbal abuse and bullying than the highly extrinsically satisfied. Similar findings were found among those classified as less intrinsically satisfied. However, no significant difference was found between those who were highly satisfied and those who were less satisfied about being bullied or harassed when the assessment of their level of satisfaction was based upon who they were bullied by, whether it be their patients, their patients’ relatives or other staff (Table 4).

### 3.5. Risk factors for job satisfaction

#### 3.5.1. Physicians

In the final models, three variables—employment sector, working a shift pattern (i.e. being on call between 6 pm and 7 am) and worry about WPV—emerged as significant correlates of both intrinsic and extrinsic job satisfaction. For intrinsic job satisfaction, the adjusted odds ratios for being “worried” and “a bit worried” about WPV came in at 0.137 (95% CI: 0.033–0.559) and 0.217 (95% CI: 0.065–0.716), respectively. This implies that those “worried” or “a bit worried” about WPV tended to be 86.3% and 78.3%, respectively, less likely to be satisfied with their jobs compared with those expressing no anxieties about WPV. Physicians who worked in private organizations (AOR = 0.072, 95% CI: 0.020–0.257) and were on call through the night (between 6pm and 7am; AOR = 0.328, 95% CI: 0.118–0.913) were 92.8% and 67.2%, respectively, more likely to be dissatisfied with their jobs than those working for public organizations (Table 5).

**Table 5:** Association between risk factors and intrinsic job satisfaction—Physicians.

	Intrinsic Satisfaction^a^^,^^b^^,^^c^				
Factors	β	SE	COR	AOR	95% CI
Employment sector					
Private organization	-2.628	0.647	0.175**	0.072**	0.020–0.257
Non-profit organization	-1.328	0.777	0.348	0.265	0.058–1.216
Public organization	Reference			1	
Working on call between 6 pm and 7 am	-1.116	0.523	0.670	0.328*	0.118–0.913
Worrying violence level					
Not worried	Reference			1	
A bit worried	-1.530	0.610	0.352*	0.217*	0.065–0.716
Worried	-1.990	0.718	0.370^+^	0.137*	0.033–0.559
Perceived stress	-0.455	0.520	0.390*	0.634	0.229–1.758
Experience with physical attack in the last 12 months	0.019	1.358	1.000	0.982	0.069–14.044
Experience with verbal abuse in the last 12 months	-0.335	0.538	0.798	1.398	0.487–4.013
Experience with being bullied/mobbed in the last 12 months	0.325	0.748	0.655	0.723	0.167–3.134
Experience with sexually harassment in the last 12 months	-0.113	1.498	0.961	1.120	0.059–21.105
Experience with racially harassment in the last 12 months	-2.189	1.457	3.000	8.928	0.514–155.102

COR = Crude odd ratio; AOR = Adjusted odd ratio. SE = Standard error.

^a^Overall model: χ2 = 35.547, p < 0.001

^b^R^2^ = 0.290 (Cox & Snell), 0.386 (Nagelkerke).

^c^Overall classification rate—70.2%.

^+^ p < 0.10;

* p < 0.05;

** p < 0.005

The final model for extrinsic satisfaction was similar to that representing intrinsic satisfaction. Working in private organizations (AOR = 0.203, 95% CI: 0.066–0.625), on a shift pattern (being on call between 6 pm and 7 am) (AOR = 0.258, 95% CI: 0.095–0.699) and worrying about WPV (AOR = 0.132, 95% CI: 0.034–0.518) emerged as significantly correlated with extrinsic satisfaction in physicians. (Table 6).

**Table 6:** Association between risk factors and extrinsic job satisfaction—Physicians.

	Extrinsic Satisfaction^a^^,^^b^^,^^c^				
Factors	β	SE	COR	AOR	95% CI
Employment sector					
Private organization	-1.596	0.574	0.437^+^	0.203*	0.066–0.625
Non-profit organization	-1.006	0.726	0.504	0.366	0.088–1.518
Public organization	Reference			1	
Working on call between 6 pm and 7 am	-1.354	0.508	0.533	0.258*	0.095–0.699
Worrying violence level					
Not worried	Reference			1	
A bit worried	-0.485	0.534	0.632	0.615	0.216–1.754
Worried	-2.022	0.696	0.296*	0.132**	0.034–0.518
Perceived stress	-0.060	0.506	0.579	0.942	0.349–2.540
Experience with physical attack in the last 12 months	-0.642	1.339	1.000	0.526	0.038–7.254
Experience with verbal abuse in the last 12 months	-0.636	0.514	1.000	1.888	0.689–5.171
Experience with being bullied/mobbed in the last 12 months	1.507	0.857	0.293^+^	0.222^+^	0.041–1.188
Experience with sexually harassment in the last 12 months	-0.433	1.332	1.000	1.542	0.113–20.971
Experience with racially harassment in the last 12 months	-1.407	1.314	3.122	4.084	0.311–53.640

COR = Crude odd ratio; AOR = Adjusted odd ratio. SE = Standard error.

^a^Overall model: χ2 = 35.547, p < .001

^b^R^2^ = 0.290 (Cox & Snell), 0.386 (Nagelkerke).

^c^Overall classification rate—70.2%

^+^ p < 0.10;

* p < 0.05;

** p < 0.005

#### 3.5.2. Nurses

Seven variables were identified as significant correlates for nurses’ intrinsic satisfaction: their 1) being from an ethnic minority (AOR = 0.164, 95% CI: 0.036–0.754); 2) working on the frontline (AOR = 0.163, 95% CI: 0.035–0.750); 3) being on call between 6pm and 7am (AOR = 0.582, 95% CI: 0.383–0.884); 4) worrying about WPV (AOR = 0.438, 95% CI: 0.275–0.697); 5) describing themselves as being under stress (AOR = 0.546, 95% CI: 0.380–0.786); 6) having been bullied in the past 12 months (AOR = 0.505, 95% CI: 0.288–0.886); and 7) working in the private sector. Nurses with all these characteristics were significantly less likely to be intrinsically satisfied in their jobs. Nurses who were working in non-profit organizations were two times more likely to have intrinsic satisfaction (AOR = 1.953, 95% CI: 1.072–3.559) than those working for public organizations (Table 7).

**Table 7:** Association between risk factors and intrinsic job satisfaction—Nurses.

	Intrinsic Satisfaction^a^^,^^b^^,^^c^				
Factors	β	E	COR	AOR	95% CI
Age Group					
20–29	-0.120	0.236	0.719	0.887	0.558–1.409
30–39	-0.376	0.244	0.610*	0.686	0.426–1.107
40 and over	Reference			1	
Identity to see themselves in workplace					
Minority	-1.805	0.777	0.612*	0.164*	0.036–0.754
Majority	Reference				
Frontline Staff	-1.816	0.780	0.122**	0.163*	0.035–0.750
Employment sector					
Private organization	-0.125	0.240	1.071	0.883	0.552–1.412
Non-profit organization	0.669	0.306	2.373**	1.953*	1.072–3.559
Public organization	Reference			1	
Working on call between 6 pm & 7 am	-0.541	0.213	0.455	0.582*	0.383–0.884
Worrying violence level					
Not worried	Reference			1	
A bit worried	-0.298	0.235	0.637*	0.743	0.468–1.178
Worried	-0.825	0.237	0.302**	0.438**	0.275–0.697
Perceived stress	-0.605	0.186	0.403**	0.546**	0.380–0.786
Experience with physical attack in the last 12 months	-0.026	0.252	0.595*	0.975	0.595–1.598
Experience with verbal abuse in the last 12 months	-0.070	0.203	0.524**	0.933	0.626–1.389
Experience with being bullied/mobbed in the last 12 months	-0.683	0.287	0.338**	0.505*	0.288–0.886
Experience with sexually harassment in the last 12 months	0.617	0.433	1.008	1.853	0.793–4.329
Experience with racially harassment in the last 12 months	0.003	0.599	0.710	1.003	0.310–3.242

COR = Crude odd ratio; AOR = Adjusted odd ratio. SE = Standard error.

^a^Overall model: χ2 = 35.547, p < .001

^b^R^2^ = 0.290 (Cox & Snell), 0.386 (Nagelkerke).

^c^Overall classification rate—70.2%.

* p < 0.05;

** p < 0.005

Six variables emerged as significant correlates of extrinsic satisfaction in nurses: their 1) being aged between 30 and 39 (AOR = 0.509, 95% CI: 0.313–0.827); 2) working on a shift pattern (AOR = 0.430, 95% CI: 0.265–0.698); 3) worrying about WPV (AOR = 0.466, 95% CI: 0.294–0.739); 4) being under stress (AOR = 0.601, 95% CI: 0.417–0.867); 5) having been verbally abused in the past 12 months (AOR = 0.647, 95% CI: 0.436–0.961); and 6) working for non-profit private organizations. Nurses meeting these descriptions were significantly less likely to be extrinsically satisfied in their jobs. Those working for non-profit private organizations were three times more likely to have extrinsic satisfaction than those working in public organizations (AOR = 3.047, 95% CI: 1.639–5.666) (Table 8).

**Table 8:** Association between risk factors and extrinsic job satisfaction—Nurses.

	Extrinsic Satisfaction^a^^,^^b^^,^^c^				
Factors	β	SE	COR	AOR	95% CI
Age Group					
20–29	-0.404	0.244	0.544*	0.667	0.414–1.077
30–39	-0.675	0.247	0.442**	0.509*	0.313–0.827
40 and over	Reference			1	
Employment sector					
Private organization	0.298	0.236	2.858**	1.347	0.848–2.140
Non-profit organization	1.114	0.316	0.806	3.047**	1.639–5.666
Public organization	Reference			1	
Shift duty	-0.844	0.247	0.334**	0.430**	0.265–0.698
Worrying violence level					
Not worried	Reference			1	
A bit worried	-0.115	0.232	0.778	0.891	0.565–1.405
Worried	-0.763	0.235	0.296**	0.466**	0.294–0.739
Perceived stress	-0.509	0.187	0.462**	0.601*	0.417–0.867
Experience with physical attack in the last 12 months	0.164	0.258	0.431**	1.178	0.711–1.952
Experience with verbal abuse in the last 12 months	-0.435	0.202	0.360**	0.647*	0.436–0.961
Experience with being bullied/mobbed in the last 12 months	-0.236	0.288	0.410**	0.790	0.449–1.389
Experience with sexually harassment in the last 12 months	-0.461	0.464	0.395*	0.631	0.254–1.567
Experience with racially harassment in the last 12 months	0.022	0.612	0.798	1.023	0.308–3.396

COR = Crude odd ratio; AOR = Adjusted odd ratio. SE = Standard error.

^a^Overall model: χ2 = 35.547, p < 0.001

^b^R^2^ = 0.290 (Cox & Snell), 0.386 (Nagelkerke).

^c^Overall classification rate—70.2%.

* p < 0.05;

** p < 0.005

## 4. Discussion

The study’s findings revealed no significant age and gender differences in both intrinsic and extrinsic job satisfaction among physicians and nurses. In this sense, our findings match those of Voltmer et al., [27]. Voltmer [27] compared job stress and job satisfaction among physicians in private practice in two European countries (Germany, n = 414; Norway, n = 340). Results showed that job satisfaction was significantly higher among Norwegian than German physicians. Cultural differences between countries, differences in weekly hours worked and the perceived effort-reward balance for physicians may contribute to this difference in level. Voltmer [27] concludes that a perceived imbalance between effort and rewards may represent a risk factor behind doctors’ physical and mental health problems.

### 4.1. Age

Nurses being aged between 30 and 39 was one of the significant correlates bearing on nurses’ extrinsic job satisfaction. Over 60% of these nurses (63.4%) had less than 10 years of clinical experience and that 96.5% of nurses categorized themselves as front-line nurses. Only a small fraction of our sample, then, (3.5%) were nurse officers or managers. Nurses with less than ten years of post-registration experience may be appointed as nurse in-charge of a clinical unit. Clinical inexperience may represent a further stress on these nurses. [32] The situation gets worse for nurses facing WPV (e.g. verbal abuse / bullying /mobbing), especially since many are likely to have to deal with the perpetrators on their own. It is not likely, in Macau’s nursing culture, that “violent incidents debriefing” was available to nurses. Nurses may thus have no one to talk with about the psychological impact WPV was having on them. They may in consequence have felt badly rewarded [37], or hampered in their promotion prospects. [38]

### 4.2. Employment sector

Working in private organizations turned out to be a significant correlate for physicians’ intrinsic and extrinsic job satisfaction. Our findings contradicted a Finnish study [39], which examined job satisfaction and well-being among 1047 male and 1522 female physicians in both public and private sectors. Heponiemi et al. [39] find physicians working in the private sector had higher levels of job satisfaction, organizational commitment and lower levels of distress compared to doctors in the public sector. Numerous studies have addressed work-related stress in physicians in hospitals [40–43] but there is only a paucity of empirical studies investigating levels of work-related stress among physicians in private practice. [44–47] Restricted professional autonomy [48, 49] may lead to unhappiness with one’s job. Job dissatisfaction may in turn add to physicians’ level of work-related stress [46, 50]. That nurses were working for non-profit organizations were also a significant correlate affecting intrinsic and extrinsic job satisfaction in nurses. Only 12% (n = 72) (i.e. ~ 1 in 8) of our nurse samples worked in this context. It is plausible that these non-profit organizations were partially subsidized by the Macau Health Bureau. With few resources and limited support from managers and colleagues, nurses may have to take care of the clients on their own for most of their time. The nurse-patient ratio would be higher in non-profits. Work overload may lead to their feeling more and more stressed [51]. Lack of career prospects, poor managerial support, low income and WPV may lead to nurses’ increased extrinsic job dissatisfaction. Low job satisfaction and an imbalance between effort and reward have been described as risk factors for symptoms of physical and mental health and illness in various occupational groups, especially nurses and physicians. [27, 52, 53]

### 4.3. On-call duty and shift work

Being on call means being ready to work between 6pm and 7am. This shift rota emerged as one of the significant risk factors affecting intrinsic and extrinsic job satisfaction among physicians. On-call duty is correlated in the study with nurses’ intrinsic job satisfaction, while shift work relates to their extrinsic job satisfaction. Our findings are in line with those Dan et al. [21] Long working hours and work strain seem to be a contributor to job dissatisfaction [19,21,27] among physicians and nurses. Due to the bureaucracy of the healthcare system, they may then have to tolerate long working hours on shift in silence. Low levels of control over their working and little decision latitude have been established as predictors for physicians’ and nurses’ dissatisfaction or burnout. [19] Physical and mental exhaustion may compromise physicians’ and nurses’ physical well-being [54], affecting the quality of care they offer [55] and, in turn, the relationship between caring professionals and patients.

It is almost certainly the case that relatively few physicians and nurses in our study would have been working during the ‘on-call’ period (i.e. 6pm and 7am). In consequence, physicians and nurses may have to deal with a large influx of patients waiting longer for shorter consultations. Patients easily project their anger, and frustration at medical and nurse professionals. Poor communication between physicians (or nurses) and patients may exacerbate tensions between healthcare providers and service recipients [21]. The recipients of care can easily lose their temper when a worse standard of provision is offered. Their impatience, together with poor staff-patient communication [56] may substantially increase the risk of verbal abuse and other forms of WPV in healthcare settings.

### 4.4. Verbal abuse and bullying

In the multivariate logistic regression, we found that verbal abuse and bullying were significantly correlated with nurses’ intrinsic and extrinsic job satisfaction. Bullying was significantly correlated with physicians’ extrinsic job satisfaction, though only to a marginal degree of significance (p < .01). In this study, 85.2% of nurses had been bullied in some form, compared with 88.5% of physicians, over the twelve months of the report period. The prevalence we found of different forms of WPV among physicians ranged from 61.5% to 96.2%. Between 43.4% to 97.5%. 43.4% of nurses had been verbally abused in the past year. The prevalence rate for bullying stated in our study was significantly higher than that reported in most European studies, which report between 10% to 15% [57].

Our overall prevalence rates of verbal abuse (53.4%), physical assault (16.1%), bullying or harassment (14.2%), and sexual harassment (4.6%) bear a close resemblance to those reported in another recent study of Macau conducted by Mai et al [58] (N = 672) (verbal abuse: 56.8%; physical assault (13%), bullying/mobbing (32.1%), sexual harassment (8.2%); this study was conducted in 2014). Using the same WPV instrument, Mai and colleagues measured WPV among physicians and nurses (N = 672). Mai’s study, however; did not examine the prevalence rates of WPV for the two different professions, making it impossible for us to compare our results for prevalence as concerns physicians and nurses separately with Mai’s findings. We found more verbal abuse and bullying than Mai’s. This discrepancy may arise from a difference of research sites, as Mai’s study was conducted primarily in one public hospital in Macau whereas ours covered multiple sites, including public, private and non-profit organizations. The nature of healthcare organizations, their staff-to-patient ratios and levels of service provision may affect prevalence estimates for WPV.

Just looking at the prevalence rates of WPV towards nurses by itself, though, our results for the incidence of these forms of abuse comes in much higher than those of a recently conducted large-scale cross-sectional study (N = 850) conducted by Cheung & Yip in Hong Kong [33]. The most common forms of WPV in Cheung & Yip’s study was verbal abuse or bullying (39.2%), followed by physical assault (22.7%) and sexual harassment (1.1%).

### 4.5. Worry about WPV

Worry about WPV emerged as a significant correlate for physicians’ and nurses’ intrinsic and extrinsic job satisfaction in this study. Although physicians and nurses experienced different forms of WPV, the prevalence rates of WPV actually came in higher among physicians (ranging from 61.5% to 96.2%) than for their nurse counterparts (ranging from 43.3% to 95.2%). Interestingly, physicians worried less about WPV than nurses (50% vs 69.1%). This situation may, perhaps, be explained by their position in medical hierarchies (which could be perceived as insulating them from the threat of malpractice suits or internal discipline), their perceived strength (suggesting that they do not face a serious threat of physical violence), and the longer duration of direct patient contact for nurses. Even though many doctors had experienced some form of WPV, they were still obliged to provide a good quality of care to their patients. Physicians, further, may not want to be seen as ‘unprofessional’ in complaining about the threat of WPV, possibly believing this would threaten their career prospects. A possible consequence is that our study underestimates prevalence rates of physicians’ worrying about WPV.

In a comparable study, Yao et al. [59] examine associations between workplace violence, general self-efficacy and occupational stress among 758 doctors in nine hospitals in Henan province, China. Results show that physicians experiencing and witnessing workplace violence were significantly more likely to be under occupational stress and less likely to be satisfied in their work. Job stress and job satisfaction of physicians are both important indicators of the quality of care offered by hospital systems. [46] Job satisfaction has been found to be correlated not only with doctors’ mental health but also with the quality of care they provide. [60]

Nurses, however, have always been working on the frontline providing direct patient care. The authors of this study report elsewhere that being directly exposed to patient care on the frontline represents a risk factor for WPV [18] in healthcare settings. To a greater degree than physicians, nurses must deal with patients and their family in day-to-day clinical encounters. This places frontline nurses at an especially high risk of WPV, compared with physicians and other health workers.

### 4.6. Ethnic minority status

Coming from an ethnic minority was a significant correlate affecting nurses’ intrinsic job satisfaction. Ninety-four (15.6%) nurses in our sample placed themselves in an ethnic minority in Macau, a percentage representing only a fraction of the nurses participating in the study. We can presume that nurses moved to Macau to seek out better jobs, working conditions and other benefits. [61] Cultural differences between Macau and their place of origin may cause difficulties for nurses coming to work in the former colony. It is possible that nurses’ working conditions differed by ethnicity, as could nurses’ perceived or actual socioeconomic statuses; and these could have led to disparities in the mental health of minority and cultural majority nurses. [62] Job satisfaction can be taken to represent a complete cognitive and affective evaluation of an individual’s working conditions. [63] Perceived differences in occupational prestige as these may disadvantage ethnic minority nurses also lie beyond the scope of this study’s observation. [64] There is also the possibility that patients expressed bias or prejudice against non-native nurses, may be because of communication barriers. Some patients could perceive non-local nurses as ‘outsiders’. Tensions between ethnic minority nurses and patients can easily eventuate in misunderstandings, verbal abuse, bullying or other forms of WPV.

### 4.7. Perceived stress

Perceived stress was a significant correlate with lower levels of intrinsic and extrinsic job satisfaction in nurses. As the Macau population has grown, nurses have taken on an increased, and ever complex workload, forcing them to shoulder a great physical and emotional burden of stress while feeling lower job satisfaction and levels of reward. [37, 65] Shift work, job satisfaction, and symptoms of stress were all significantly correlated with WPV for nurses. [23]

Violence impacts not only the individual affected but also her or his employer, as exposure may lead to poor employee morale, low job satisfaction, and high rates of turnover [23] and poor retention rates. [22, 37] A better work atmosphere with less of a physical burden placed on professionals, better relations with colleagues, a greater measure of professional autonomy and of control over clinical work, together with better support from senior managers [66] and shorter work hours may lead to higher job satisfaction among physicians and nurses. [40]

There is evidence that training programmes in the management of workplace violence (MOV) [67] are effective in increasing medical professionals’ competency, attitudes, knowledge and practical de-escalation skills when faced with situations with patients that threaten violence.

## 5. Limitations

This study is marked by some limitations. First, cross-sectional data cannot establish causal relationships between job satisfaction and WPV. Second, there is a risk of sampling bias as the nature of the study may disproportionately draw those who have exposed to WPV in the preceding year to participate. Equally, those medical professionals who have not been subject to WPV and have little interest in the subject may choose not to participate. This sampling bias might lead to over-or-underestimation—though more probably the former—of the actual incidence of WPV in Macau healthcare settings. Notwithstanding these limitations, our sampling frame includes the key healthcare organizations in Macau. It is possible that stratified random sampling might be more representative of the experience of all medical professionals in Macau, and that this method would boost the generalizability of our findings. All the same, the findings of this study should provide a starting point for future work on WPV and its prevention.

## 6. Conclusions

WPV remains a significant concern in healthcare settings in Macau. The existing empirical literature stresses physical violence, making this particular manifestation of WPV the focus of workplace intervention. What seems to be less recognized is the psychological impact of non-physical forms of violence like verbal abuse and bullying. Our findings clearly indicate that non-physical violence plays a pivotal role in medical-related professionals’ job satisfaction and mental health. Exposing to both physical and non-physical violence represents a significant hazard in the healthcare setting which warrants further research, not least to put in place effective prevention initiatives.
